# Translocator protein positron emission tomography as a biomarker for posttraumatic epilepsy and behavioral comorbidities following traumatic brain injury

**DOI:** 10.1002/epi.70410

**Published:** 2026-07-28

**Authors:** Mohit Javalgekar, Peravina Thergarajan, Juliana Silva, Emma Braine, Robert Brkljača, Pablo M. Casillas‐Espinosa, Stefanie Dedeurwaerdere, Mohammad Haskali, Usama Kamil, Terence J. O'Brien, Nigel C. Jones, David K. Wright, Bianca Jupp, Lucy Vivash, Idrish Ali

**Affiliations:** ^1^ Department of Neuroscience, School of Translational Medicine Monash University Melbourne Victoria Australia; ^2^ Monash Biomedical Imaging Clayton Victoria Australia; ^3^ University of Antwerp Antwerp Belgium; ^4^ Peter MacCallum Cancer Centre Melbourne Victoria Australia; ^5^ Department of Neurology Alfred Hospital Melbourne Victoria Australia

**Keywords:** biomarker, inflammation, neuroimaging, posttraumatic epilepsy, traumatic brain injury

## Abstract

**Objective:**

Traumatic brain injury (TBI) induces widespread and persistent neuroinflammation, which is increasingly recognized as a key contributor to chronic sequelae including posttraumatic epilepsy (PTE), cognitive deficits, and neuropsychiatric disturbances. However, reliable biomarkers for identifying individuals at risk for these long‐term outcomes remain limited. In this study, we evaluated in vivo positron emission tomography (PET) imaging of the 18‐kDa translocator protein (TSPO) as a predictive biomarker of epileptogenesis and neurobehavioral dysfunction in a rat model of moderate–severe TBI.

**Methods:**

Adult rats received lateral fluid percussion injury (*n* = 61) or sham surgery (*n* = 14). TSPO PET was performed using [^18^F]‐PBR06 at 1 and 4 weeks postinjury. Long‐term behavioral outcomes were assessed at 5–6 months post‐TBI, followed by continuous 4‐week video‐electroencephalographic monitoring during 7^th^ month to identify epileptic rats.

**Results:**

TBI rats demonstrated robust and sustained increases in TSPO binding across multiple brain regions at both time points compared with sham animals (*p* < .001). At 4 weeks postinjury, however, TSPO binding in the cortex, hippocampus, and thalamus was significantly lower in rats that later developed epilepsy (PTE^+^) than in those that did not (PTE^−^; *p* < .05), with receiver operating characteristic (ROC) analysis showing good predictive performance (area under the curve [AUC] = .76, *p* < .01). Behavioral testing identified deficits in spatial learning, memory, and open‐field activity in TBI rats (*p* < .05). Data‐driven stratification identified behaviorally impaired (TBI‐BI^+^) and unimpaired (TBI‐BI^−^) subgroups, with cortical TSPO moderately distinguishing impaired animals (AUC ≈ .7). Among impaired rats, those with multidomain behavioral deficits (*n* = 33) showed elevated thalamic TSPO compared to those with a single impairment (*p* = .032), with ROC analysis confirming discrimination (AUC = .72, *p* = .018).

**Significance:**

Our findings support TSPO PET imaging as a promising early biomarker for predicting PTE and neurobehavioral outcomes following TBI.

**Key points**

- TSPO PET detects early and persistent neuroinflammation after moderate–severe TBI.
- Reduced subacute TSPO binding predicts posttraumatic epilepsy, distinguishing PTE^+^ from PTE^−^ rats.
- Regional TSPO binding relates to long‐term behavioral impairment following TBI.
- Elevated thalamic TSPO binding identifies animals with multidomain behavioral deficits.

## 1 INTRODUCTION

Traumatic brain injury (TBI) occurs when physical trauma to the head or neck causes functional and pathological changes in the brain.^1^ TBI is a leading cause of death and chronic disability worldwide^2^ and is associated with a range of chronic sequelae, including cognitive impairment,^3^ neuropsychiatric disturbances,^4^ and posttraumatic epilepsy (PTE), which substantially compromise quality of life and recovery. PTE is defined by the occurrence of recurrent unprovoked seizures developing at least 1 week following TBI^5^ and may emerge months to years following the initial insult. PTE represents one of the most severe complications of TBI and accounts for 5% of all cases of epilepsy and 20% of all symptomatic epilepsy.^6^

The development of PTE is preceded by a latent or asymptomatic period during which complex molecular, inflammatory, and structural changes occur in the injured brain.^7^ Although the latent period also presents a potential window for preventive therapeutic intervention, identifying patients at risk of developing PTE remains challenging due to the lack of reliable predictive biomarkers.^8^ A better understanding of the mechanisms underlying posttraumatic epileptogenesis could lead to the identification of biomarkers and potential preventive antiepileptogenic treatments.

Emerging evidence suggests that neuroinflammation plays a key role in PTE pathophysiology, with multiple inflammatory mediators linked to epileptogenesis.^9^, ^10^ Neuroinflammation, characterized by glial activation and cytokine release, can disrupt ion channel and neurotransmitter signaling and alter synaptic plasticity, promoting network hyperexcitability and epileptogenesis.^11^ Noninvasive neuroimaging techniques, such as positron emission tomography (PET) using radiotracers that target the 18‐kDa translocator protein (TSPO), offer a noninvasive approach for quantifying neuroinflammation in vivo. TSPO, located on the outer mitochondrial membrane, is upregulated upon microglial and astrocytic activation during neuroinflammation.^12^ TSPO PET is increasingly used to characterize neuroinflammation across multiple neurological disorders.^13^ Elevated TSPO levels measured by PET reflect inflammation‐related pathology and have been used to monitor disease progression, stratify patient subgroups, and evaluate anti‐inflammatory or immunomodulatory therapies.^14^ Increasing evidence also supports the application of TSPO PET in epilepsy, where neuroinflammatory mechanisms are integral to seizure generation and disease progression. Increased uptake of TSPO specific radiotracers has been consistently observed in both preclinical models and clinical populations with temporal lobe epilepsy (TLE).^15^, ^16^ In individuals with TLE, increased TSPO PET signal is observed in brain regions ipsilateral to the seizure focus compared with healthy controls, indicating localized neuroinflammation within the epileptogenic network.^17^ Importantly, subsequent studies have shown that TSPO PET signal is not strictly confined to the primary epileptogenic focus, but may also be detected in contralateral mirror regions and other shared epileptic networks, including conditions such as malformations of cortical development, suggesting more widespread neuroinflammatory involvement.^18^, ^19^, ^20^ Furthermore, TSPO PET can localize seizure foci even in magnetic resonance imaging (MRI)‐negative, drug‐resistant cases, underscoring its sensitivity beyond conventional structural imaging.^21^ Preclinical studies further reveal marked TSPO upregulation during both the latent and chronic phases of epileptogenesis.^22^, ^23^ Early TSPO signal changes have been shown to predict later seizure burden, underscoring its potential as a biomarker of epileptogenic progression.^22^

Beyond epilepsy, neuroinflammation is also implicated in long‐term neurological and behavioral impairments following TBI through glial activation and cytokine release that disrupt neural circuits.^24^ These changes contribute to persistent cognitive and affective deficits, including impairments in learning, memory, and mood regulation as reported by previous studies,^25^, ^26^ suggesting that sustained neuroinflammation may link TBI, PTE, and neurobehavioral comorbidities. Therefore, to investigate the predictive potential of TSPO PET for epilepsy and behavioral impairments following TBI, we used the lateral fluid percussion injury (LFPI) rat model, which develops PTE in ~20%–30% of animals.^27^ TSPO PET imaging was performed at 1 and 4 weeks postinjury, followed by behavioral testing at 5–6 months and video‐electroencephalographic (EEG) monitoring at 6 months to identify animals that developed epilepsy.

## 2 MATERIALS AND METHODS

### 2.1 Overall study design

Male and female Sprague Dawley rats underwent either LFPI or sham surgery using standard procedures. At 1 week and again at 4 weeks postinjury, each animal underwent MRI and TSPO PET imaging using an intravenous bolus of the [^18^F]‐PBR06. Behavioral assessments were conducted at 5–6 months postinjury. At 6 months, continuous video‐EEG monitoring was performed for 4 weeks to detect spontaneous recurrent seizures and identify animals that developed PTE. Animals were euthanized at 7 months for tissue collection.

### 2.2 Experimental animals

Rats were purchased from Monash Animal Research Services. At the time of injury, the rats were 11–12 weeks old (387 ± 32 g males, 225 ± 16 g females). Rats were housed under standard laboratory conditions with ad libitum food and water. All experimental procedures were approved by the Alfred Medical Research and Education Precinct Animal Ethics Committee (E/2078/2021/M).

### 2.3 Lateral fluid percussion injury

Following procurement, rats (*n* = 112) were randomly assigned to either the TBI group (*n* = 95; 49 males, 46 females) or the sham‐surgery group (*n* = 17; 10 males, seven females). Of the TBI group, 28 rats died immediately or reached the prescribed humane endpoint within the first week postinjury. An additional nine rats (six TBI, three sham) were excluded due to loss of head implant or surgical complications. The final cohort included 61 LFPI rats (30 males, 31 females) and 14 sham‐operated controls (nine males, five females). Briefly, as described previously,^27^ rats were anesthetized with isoflurane (5% induction, 2%–3% maintenance in 100% O_2_ at 1 L/min) and received preoperative analgesia consisting of buprenorphine (.05 mg/kg sc, Tasmanian Alkaloids) and carprofen (5 mg/kg sc, Pfizer). A 5‐mm craniotomy (anteroposterior: −4.5 mm, mediolateral: 2.5 mm relative to bregma) was performed to expose the dura. A Luer‐Lock cap was fixed around the craniotomy, filled with sterile saline and connected to the fluid percussion injury device (AmScien Instruments). A moderate–severe injury (~3 atm) was induced by releasing the pendulum to deliver a saline pulse onto the dura. Only craniotomy was performed on sham controls. Rats received a subcutaneous saline bolus (10 mL/kg) for hydration before returning to their home cages. Postoperative analgesia was administered at 24 and 48 h and thereafter as required based on pain assessment.

### 2.4 PET and MRI neuroimaging

T2‐weighted MRI scans were first acquired on a Bruker 9.4‐T BioSpec system using a two‐dimensional rapid acquisition with relaxation enhancement (RARE) sequence (repetition time = 3400 ms, RARE factor = 8, effective echo time = 45 ms, slice thickness = .8 mm, field of view = 30 × 30 mm^2^, matrix = 256 × 256, resolution = 117 × 117 μm^2^). For PET imaging, the radiotracer [^18^F]‐PBR06 was synthesized with radiochemical purity > 95% as previously described.^28^ Rats were anesthetized with isoflurane (induction: 5% in 1 L/min O_2_; maintenance: 1.5%–2% in 1 L/min O_2_) and maintained at 37°C throughout the scanning period. Rats received a tail vein bolus injection of [^18^F]‐PBR06 (mean dose = 17.93 MBq, range = 8.10–31.67 MBq) and underwent a 60‐min PET acquisition in a nanoScan PET–computed tomography (CT) scanner (Mediso Imaging Systems). A CT scan (80 kV/500 μA, 10 min) was acquired for attenuation correction. PET images were reconstructed from the 40–60‐min postinjection window, corresponding to the stabilization of tissue uptake, therefore improving target to background contrast for TSPO quantification. CT images were coregistered with MRI scans, and volumes of interest (VOIs) from both hemispheres were delineated on MRI using PMOD 4.0 based on a standard rat brain atlas. All VOIs were reviewed and verified by experienced reviewers for anatomical accuracy and consistency.

Regional uptake was expressed as percentage injected dose (%ID) calculated by normalizing the activity concentration in each VOI (kBq/mL) to the administered dose. It was chosen as primary PET outcome parameter to minimize potential variability associated with sex‐related differences in body weight and body composition including fat to muscle ratio, which may influence standardized uptake value measurements in mixed sex cohorts.^29^ All image processing and quantification were performed by an investigator blinded to epilepsy outcomes.

### 2.5 Behavioral assessments

From 5 to 6 months post‐TBI, all rats underwent a series of well‐established behavioral assessments to evaluate anxietylike behavior (elevated plus maze and open field test), learning and memory (Morris water maze and novel object recognition test), and depressionlike behavior (sucrose preference test and forced swim test) as described previously.^30^ Due to its moderately stressful nature, the forced swim test was performed after video‐EEG monitoring at 7 months postinjury immediately prior to euthanasia. For detailed behavioral protocols, see Supporting Information.

### 2.6 Electrode implantation surgery

At 6 months post‐TBI, rats were implanted with subdural screw electrodes (Plastics One) under isoflurane anesthesia as previously reported.^31^ Preoperative treatment included local bupivacaine (1 mg/kg, Pfizer) and a combination of buprenorphine (.05 mg/kg sc, Tasmanian Alkaloids) and carprofen (5 mg/kg sc, Pfizer). After a midline scalp incision, six electrodes were placed (frontal ×2, parietal ×2, occipital ×2, the latter serving as ground and reference), along with one anchoring screw for stability. The assembly was secured with dental acrylate (Vertex). Postoperative analgesia was administered as described in the LFPI surgery section above. Rats were allowed to recover for 1 week prior to EEG recording.

### 2.7 Video‐EEG monitoring and seizure detection

Following recovery from electrode implantation surgery, rats were connected to a six‐channel EEG acquisition system (Neuvo, Compumedics). Continuous EEG and synchronized video recordings were obtained for 1 month at a sampling rate of 500 Hz using Profusion 6 software (Compumedics). Seizures were detected using the semiautomated software Assyst,^32^ with the investigators blinded to the experimental condition, and confirmed by combined analysis of EEG recordings and video data. Epilepsy outcomes included seizure occurrence, frequency (seizures per month), and duration.

### 2.8 Statistics

All statistical analyses were performed using GraphPad Prism (v10.3.0). Data normality was assessed using the Shapiro–Wilk test. Depending on normality statistics, either parametric or nonparametric statistical analyses were applied as appropriate. Group differences in PET imaging data and Morris water maze training performance were analyzed using repeated‐measures two‐way analysis of variance (ANOVA) followed by Sidak post hoc test. Neurobehavioral outcomes were analyzed using the Kruskal–Wallis test with Dunn multiple‐comparisons test. Principal component analysis (PCA) was used to explore relationships between PET imaging and neurobehavioral measures. The predictive value of PET imaging markers for epilepsy development was assessed using receiver operating characteristic (ROC) analysis, with area under the curve (AUC) reported. Behavioral impairment in TBI rats was determined using a data‐driven cut‐point analysis with sham rats as unimpaired controls.^33^ Optimal thresholds were identified using the cutpointr package in R^34^ by maximizing the sum of sensitivity and specificity. For this analysis, PTE^+^ and PTE^−^ rats were combined into a single TBI group and compared with sham controls. Imaging outcomes were then compared between these subgroups.

## 3 RESULTS

### 3.1 Occurrence of PTE

Spontaneous recurrent seizures were detected during 1 month of continuous video‐EEG monitoring starting 6 months postinjury (Supporting Information, Figure 1). Eleven of 61 TBI rats (18%) developed spontaneous seizures (mean = 2.4 seizures, mean duration = 29 s). These animals were classified as PTE^+^ group for all subsequent analyses. The remaining 50 TBI rats (82.0%) showed no spontaneous seizures or epileptic discharges and composed the PTE^−^ group. No seizures or epileptic discharges were observed in sham‐injured rats. Sex‐specific analysis showed that the proportion of animals developing epilepsy did not differ significantly between males and females (Fisher exact test, *p* = .335; Figure S2A). Furthermore, mean seizure duration was not significantly different between male and female epileptic animals (male: 27.64 ± 3.5 s; female: 31.90 ± 14.42 s; Mann–Whitney *U*‐test, *p* = .344; Figure S2B).

**FIGURE 1:**
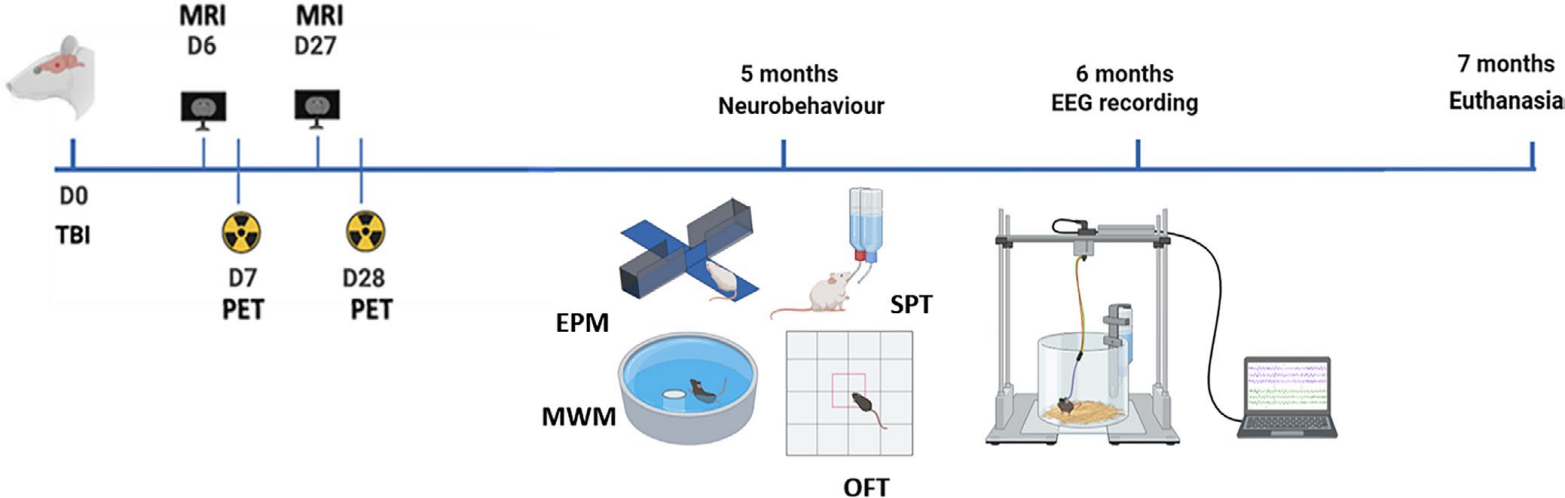
Study design showing experimental timeline. D, day; EEG, electroencephalographic; EPM, elevated plus maze test; MRI, magnetic resonance imaging; MWM, Morris water maze test; OFT, open field test; PET, positron emission tomography; SPT, sucrose preference test; TBI, traumatic brain injury.

### 3.2 TSPO PET distinguishes PTE and non‐PTE rats following TBI

[^18^F]‐PBR06 uptake was significantly higher in TBI rats compared to shams, indicating sustained TSPO upregulation (*p* < .05, two‐way repeated measures ANOVA; Figure 2B–F, Table 1). However, TSPO binding evolved differently in rats that later developed epilepsy. By 4 weeks post‐TBI, PTE^+^ rats showed reduced %ID relative to PTE^−^ rats in the cortex (*p* = .038), hippocampus (*p* = .015), and thalamus (*p* = .017) despite comparable levels at 1 week. TSPO signal also decreased from week 1 to week 4 in several regions of TBI animals (main effect of time, *p* < .05), indicating a general decline in postinjury inflammation. A time × group interaction was observed in the thalamus (*p* = .035), driven by a trend toward increased uptake over time in PTE^−^ rats only. ROC analysis of %ID in the cortex, hippocampus, and thalamus at 4 weeks post‐TBI revealed a significant distinction between PTE^+^ and PTE^−^ rats, with an AUC of .76 for each region (*p* < .01; Figure 2G). Further stratification of PTE^+^ rats based on seizure clustering (≥3 seizures/24 h, *n* = 3) revealed no differences in regional [^18^F]‐PBR06 uptake compared with nonclustering animals (*n* = 8; Figures S2C and S3). Detailed ANOVA results are provided in Table S1.

**FIGURE 2:**
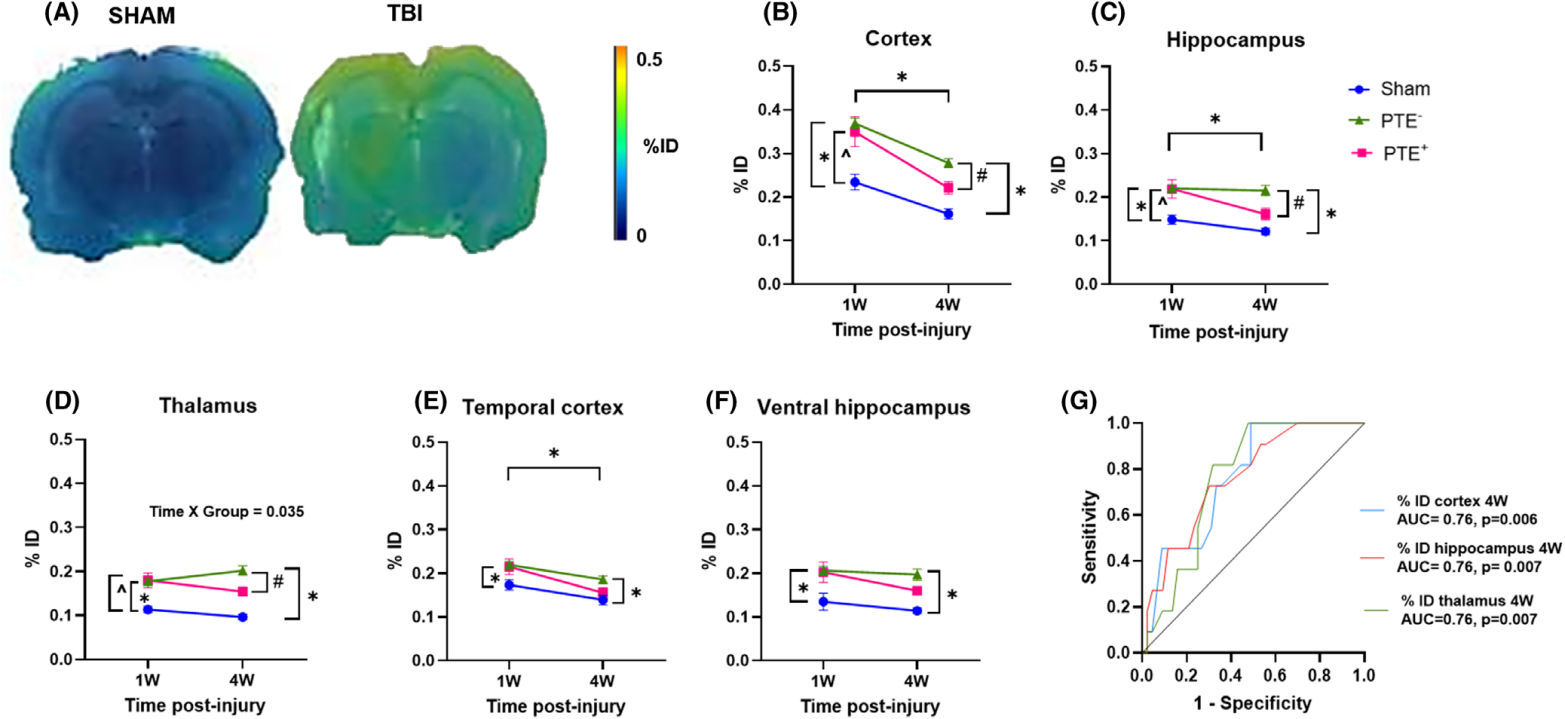
Regional [^18^F]‐PBR06 uptake values after traumatic brain injury (TBI) and their predictive value for epilepsy development. (A) Representative images of [^18^F]‐PBR06 uptake in sham versus TBI animals at 4 weeks postinjury. (B–F) Comparison of positron emission tomography percentage injected dose (ID) values across (B) cortex, (C) dorsal hippocampus, (D) thalamus, (E) temporal cortex, and (F) ventral hippocampus at 1 week and 4 weeks postinjury. (G) Receiver operating characteristic curves illustrating the predictive potential of %ID for epilepsy development. Data are presented for sham (*n* = 14), TBI without epilepsy (posttraumatic epilepsy [PTE^−^], *n* = 50), and TBI with epilepsy (PTE^+^, *n* = 11) groups. Statistical analyses were performed using two‐way analyses of variance followed by Sidak multiple‐comparisons test. *p < .05, sham versus PTE^−^; ^*p* < .05, sham versus PTE^+^; #*p* < .05, PTE^−^ versus PTE^+^. AUC, area under the curve; W, weeks.

**TABLE 1:** Regional ^18^F‐PBR06 uptake (%ID) values in experimental animals.

Group	Sham		PTE^−^		PTE^+^	
	1 week	4 weeks	1 week	4 weeks	1 week	4 weeks
Cortex	.23 ± .017	.16 ± .011	.36 ± .011*	.27 ± .009*	.35 ± .034**	.22 ± .014***
Dorsal hippocampus	.14 ± .010	.12 ± .007	.22 ± .007*	.21 ± .011*	.21 ± .020**	.16 ± .013***
Thalamus	.11 ± .007	.09 ± .006	.17 ± .006*	.20 ± .010*	.18 ± .017**	.15 ± .009***
Temporal cortex	.17 ± .011	.13 ± .010	.21 ± .008*	.18 ± .007*	.21 ± .017	.15 ± .012
Ventral hippocampus	.13 ± .019	.11 ± .006	.20 ± .009*	.19 ± .012*	.20 ± .023	.16 ± .009

*Note*: Regional ^18^F‐PBR06 binding (%ID) in sham, PTE^−^, and PTE^+^ rats at 1 and 4 weeks after traumatic brain injury. Table shows %ID measured in the cortex, dorsal hippocampus, thalamus, temporal cortex, and ventral hippocampus across experimental groups (sham, PTE^−^, and PTE^+^) at 1 week and 4 weeks postinjury. Values are presented as mean ± SEM.

Abbreviations: %ID, percentage injected dose; PTE, posttraumatic epilepsy.

* *p* < .05, sham vs. PTE^−^.

** *p* < .05, sham vs. PTE^+^.

*** *p* < .05, PTE^−^ vs. PTE^+^.

No significant sex‐related differences were observed in TSPO PET measures between male and female animals across experimental groups (*p* > .05). Correlation analyses showed significant negative associations between 1‐week TSPO %ID values across relevant brain regions and mean seizure duration (*r* ≈ .8; Figure S6). Postmortem hippocampal TSPO mRNA expression did not differ significantly between PTE^+^, PTE^−^, and sham animals. Detailed regional correlation analyses and hippocampal TSPO mRNA expression data are provided in Supporting Information (Figure S7).

### 3.3 Behavioral deficits do not relate to PTE development after TBI

An overall group effect was observed for spatial learning performance in the Morris water maze training phase (two‐way repeated‐measures ANOVA, main effect of group: *F*_2, 72_ = 10.21, *p* < .001; Figure 3A). Post hoc analysis showed significantly longer escape latencies in PTE^−^ rats compared with sham controls during training days 2–6, whereas differences between PTE^+^ and sham rats did not reach statistical significance. Similarly, an overall group difference was also observed for total distance traveled during the training phase (*p* < .001; Figure 3B). Post hoc analysis revealed significant differences between sham and PTE^−^ rats across training days 1–6, whereas a significant difference between sham and PTE^+^ rats was observed only on day 1 (*p* = .014). In the probe trial, PTE^−^ rats spent significantly less time in the target quadrant than shams (*p* = .009, Kruskal–Wallis test), and both PTE^+^ and PTE^−^ rats required longer to reach the former platform location (*p* = .003), indicating impaired spatial memory (Figure 3C,D). No differences were observed between PTE^+^ and PTE^−^ rats in probe test measures (*p* > .05).

**FIGURE 3:**
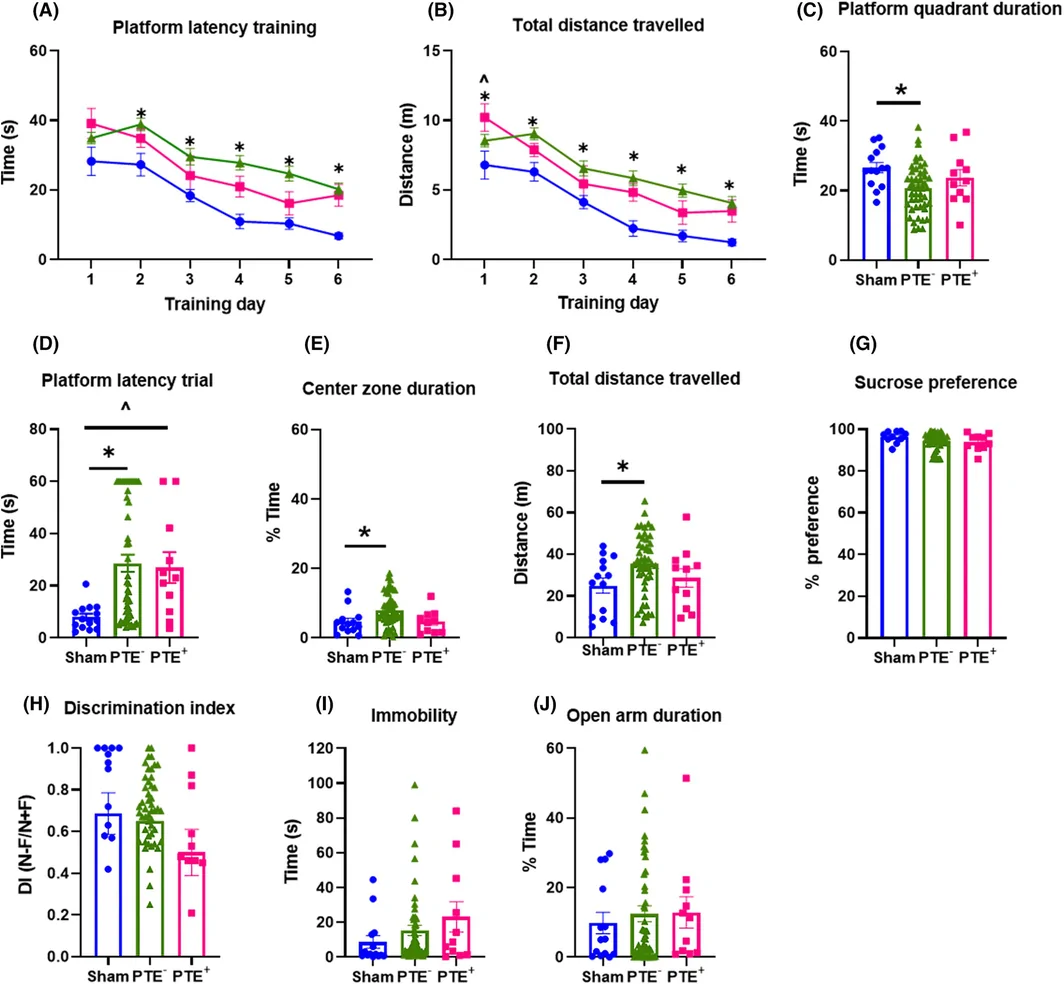
Behavioral outcomes following traumatic brain injury across cognitive and affective domains. (A) Latency to locate the platform in the Morris Water maze test. (B) Total distance traveled. (C) Duration in the platform quadrant during probe trial. (D) Latency to locate the platform on probe trial. (E) Percentage time spent exploring the center zone of open field. (F) Total distance traveled in open field. (G) Percentage sucrose preference. (H) Immobility duration in forced swim test. (I) Discrimination index (DI = [*N* − *F*] / [*N* + *F*]), where *N* = time exploring the novel object and *F* = time exploring the familiar object. (J) Percentage time spent exploring the open arm of plus maze. Error bars represent mean and SEM. *p < .05, sham versus posttraumatic epilepsy (PTE)^−^; ^*p* < .05, sham versus PTE^+^.

In the open field test, PTE^−^ rats spent significantly more time in the center zone (*p* = .025) and traveled a greater distance than sham controls (*p* = .016), whereas PTE^+^ and PTE^−^ rats did not differ from each other (Figure 3E,F). No group differences were observed in sucrose preference, forced swim, novel object recognition, or elevated plus maze tests (Figure 3G–J; all *p* > .05). Cut‐point analysis classified TBI rats as behaviorally impaired (TBI‐BI^+^) or nonimpaired (TBI‐BI^−^) across cognitive, anxietylike, and depressionlike domains (Figure S4). Sex‐specific analysis did not indicate significant differences in behavioral outcomes between male and female animals.

Contingency analysis showed no differences in the proportion of PTE^+^ and PTE^−^ rats within cognitively impaired and nonimpaired groups (Figure S5A). Similarly, the proportion of PTE^+^ rats exhibiting anxietylike or disinhibitory behavior (open field test) or depressionlike behavior (forced swim test) did not differ between TBI‐BI^+^ and TBI‐BI^−^ groups (Fisher exact test, all *p* > .05; Figure S5B,C).

### 3.4 TSPO binding predicts behavioral impairments following TBI

Using a cut‐point threshold of 4.68% time spent in the center zone of the open field test, 72.1% of TBI rats (44/61) were classified as TBI‐BI^+^, reflecting disinhibitory behavior (Figure 4A,B). TBI‐BI^+^ rats showed significantly higher cortical [^18^F]‐PBR06 %ID compared with TBI‐BI^−^ rats (main effect of group: *F*_1, 55_ = 6.20, *p* = .015), accompanied by a significant main effect of time in the cortex (*p* < .001; Figure 4C), and a trend toward higher binding in the thalamus (*F*_1, 55_ = 3.99, *p* = .050; Figure 4E). At 4 weeks post‐TBI, post hoc analysis showed a trend toward increased cortical %ID in TBI‐BI^+^ rats (*p* = .061). ROC analysis of cortical %ID distinguished TBI‐BI^+^ and TBI‐BI^−^ rats with an AUC of .71 (*p* = .014; Figure 4H). [^18^F]‐PBR06 %ID did not differ significantly between TBI‐BI^+^ and TBI‐BI^−^ rats in any of the examined regions when stratified by cognitive or depressionlike behaviors (*p* > .05; Figure S4). Thirty‐three rats exhibited deficits across all three behavioral tests based on cut‐point analysis and were classified as TBI‐BI^multi+^ (Figure 5A), whereas 15 animals showed impairment in a single behavioral domain and were designated as TBI‐BI^single+^. There was a trend toward a group difference in cortical %ID between TBI‐BI^multi+^ and TBI‐BI^single+^ rats (*p* = .069; Figure 5B). A significant group difference was observed for thalamic binding (*p* = .018), with post hoc analysis revealing significantly elevated binding in TBI‐BI^multi+^ rats at 4 weeks postinjury compared to TBI‐BI^single+^ rats (*p* = .032; Figure 5D). PCA of combined PET and behavioral data separated TBI‐BI^multi+^ and TBI‐BI^single+^ rats into two distinct clusters (Figure 5G). ROC analysis of thalamic %ID at week 4 distinguished TBI‐BI^+^ and TBI‐BI^−^ rats with an AUC of .72 (*p* = .018; Figure 5H).

**FIGURE 4:**
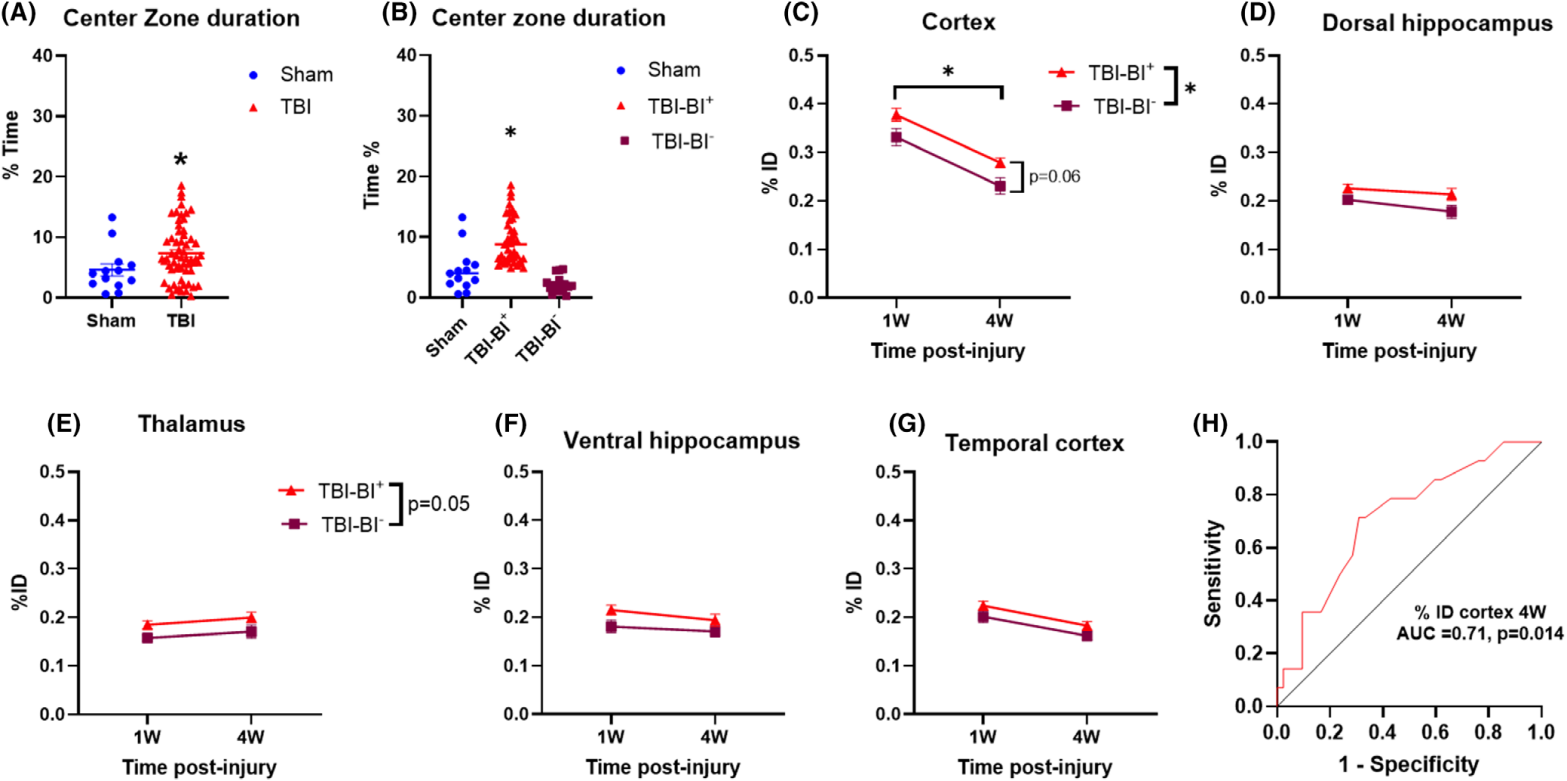
Predictive value of translocator protein (TSPO) positron emission tomography (PET) for identifying behavioral impairment in traumatic brain injury (TBI) rats. Prediction of behavioral impairment by subacute TSPO PET is shown. (A, B) Comparison of percentage time in the center zone during open field test. (C–G) Comparison of TSPO percentage injected dose (%ID) between behaviorally impaired (TBI‐BI^+^; *n* = 44) and behaviorally nonimpaired (TBI‐BI^−^; *n* = 17) rats based on time spent in center zone of open field. (H) Receiver operating characteristic curve showing the prediction of TBI‐BI^+^ animals using cortical TSPO PET binding. *p < .05. AUC, area under the curve; W, weeks.

**FIGURE 5:**
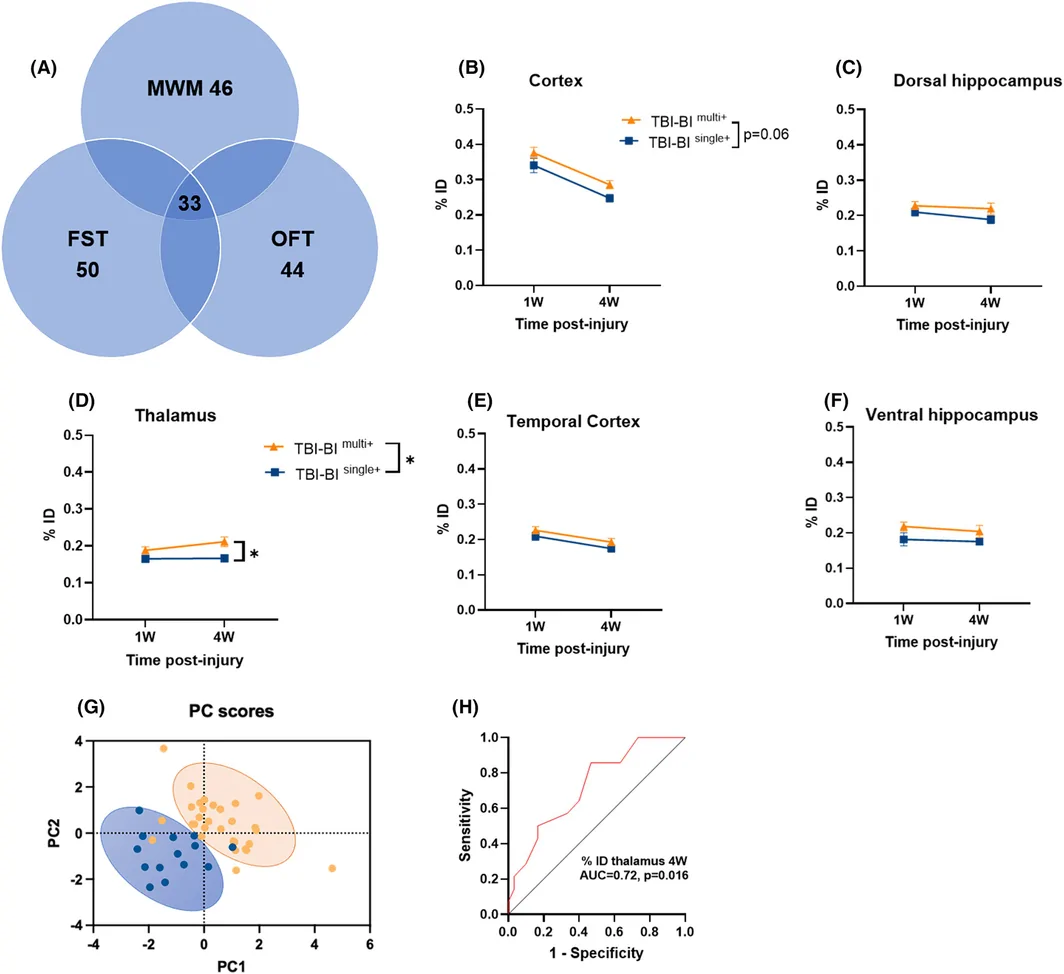
Predictive value of translocator protein (TSPO) positron emission tomography (PET) for identifying multidomain behavioral impairment in traumatic brain injury (TBI) rats. (A) Venn diagram illustrating the distribution of TBI rats exhibiting behavioral impairments across cognitive, anxietylike, and depressionlike domains. Cut‐point analysis identified 46, 44, and 50 rats as impaired in the Morris water maze (MWM), open field test (OFT), and forced swim test (FST), respectively. Numbers within circles denote domain‐specific impairments and are not additive, as individual rats may be impaired in multiple domains. Thirty‐three rats showed impairments across all three domains (TBI‐BI^multi+^), whereas 15 rats exhibited a single behavioral impairment (TBI‐BI^single+^). (B–F) Comparison of TSPO percentage injected dose (%ID) between TBI‐BI^single+^ and TBI‐BI^multi+^ groups. (G) Principal component (PC) analysis integrating PET imaging and behavioral data. (H) Receiver operating characteristic curve showing prediction of multiple behavioral impairment. Data are presented as mean ± SEM. *p < .05. AUC, area under the curve; W, weeks.

## 4 DISCUSSION

Using TSPO PET imaging in the LFPI rat model, we investigated neuroinflammation as a predictive biomarker of PTE and behavioral outcomes following TBI. Rats that later developed PTE exhibited distinct neuroinflammatory profiles, characterized by significantly lower [^18^F]‐PBR06 uptake in the cortex, dorsal hippocampus, and thalamus at 4 weeks postinjury, compared with nonepileptic TBI rats. ROC analysis confirmed that TSPO PET signal predicted PTE development, supporting its potential utility as a biomarker for early risk stratification. Furthermore, TSPO PET was also able to predict the development of some of the behavioral impairments observed as a chronic consequence of TBI.

### 4.1 TSPO as biomarker for PTE

Consistent with previous studies,^35^, ^36^, ^37^ [^18^F]‐PBR06 binding was increased in TBI rats across brain regions compared to sham rats, indicating enhanced glial activation and neuroinflammation following injury. However, a key and somewhat unexpected finding was that rats that later developed epilepsy showed relatively reduced TSPO binding at 4 weeks post‐TBI compared to those that did not develop seizures. This finding aligns with previous studies indicating that TSPO expression during epileptogenesis may predict seizure outcome. For example, in the post‐kainic acid status epilepticus (SE) rat model of TLE, TSPO binding measured 2 weeks after SE was lower in animals that later developed frequent spontaneous seizures compared with those that developed only sporadic seizures.^22^ In the same study, however, autoradiographic analysis at 12 weeks post‐SE, after epilepsy onset, revealed a positive association between TSPO binding and seizure frequency.^22^ Because EEG monitoring was not performed at the 1‐ and 4‐week imaging time points, the temporal relationship between early TSPO PET alterations and the onset of spontaneous seizures could not be directly established. However, consistent with our paradoxical finding linking lower TSPO signals to chronic PTE, 1 week TSPO %ID values within the PTE cohort demonstrated a significant negative correlation with mean seizure duration. This complexity is further reflected at the tissue level, where postmortem hippocampal TSPO mRNA expression did not differ between epileptic and nonepileptic TBI animals, highlighting the highly dynamic and nonlinear nature of TSPO expression throughout epileptogenesis. Consequently, the translational utility of TSPO PET may lie in monitoring the temporal evolution of neuroinflammation rather than relying on a single cross‐sectional measurement. Consistent with this, a significant time × group interaction was observed in the thalamus, driven by increasing TSPO uptake over time in PTE^−^ animals but not in PTE^+^ animals. This finding supports the idea that early post‐TBI imaging followed by a later scan may be more informative than a single cross‐sectional measurement and could help identify individuals at increased risk of PTE development.

Given the dynamic nature of neuroinflammation during epileptogenesis, the higher [^18^F]‐PBR06 binding observed in PTE^−^ rats may suggest that a stronger subacute inflammatory response could be protective against epileptogenesis. Conversely, the lower TSPO PET signal in PTE^+^ rats may reflect a blunted or insufficient immune response. It is also possible that microglia in PTE^+^ rats adopt activation phenotypes that are not readily detected by TSPO PET, as TSPO expression does not differentiate between microglial phenotypes. Supporting this, a study in a rat model of acute subcortical stroke showed that [^18^F]‐DPA‐714 PET detected early microglial activation in the infarct and surrounding region but failed to capture diffuse chronic microglial activation in white matter as confirmed by immunohistochemistry.^38^ Furthermore, in addition to microglial activation, astrocytes are important mediators of persistent neuroinflammation following TBI and contribute to gliosis, excitatory imbalance, and epileptogenesis.^39^ Previous literature has shown a complex nature of neuroinflammatory response characterized by sequential activation of microglia followed by reactive astrocytes, with astrocytic reactivity contributing to enhanced seizure susceptibility during epileptogenesis.^40^ However, TSPO PET primarily reflects microglial activation and may incompletely capture astrocytic reactivity, representing a limitation of TSPO imaging as a comprehensive marker of neuroinflammation. Complementary imaging approaches targeting astrocytic activation, including myo‐inositol‐based techniques such as myo‐inositol chemical exchange saturation transfer imaging may provide important insight into glial dynamics during posttraumatic epileptogenesis.^41^

Additionally, systemic or metabolic factors such as competition with endogenous TSPO ligands (e.g., cholesterol) may also influence PET signal.^42^ This is notable because cholesterol is elevated in rats with PTE^43^ and has an established role in neuronal hyperexcitability and seizure generation.^42^, ^44^, ^45^ Peripheral inflammation following TBI may also reduce tracer availability in the brain by sequestering circulating radioligand.^33^ However, this explanation appears unlikely in our study, as plasma TSPO levels at 1 and 4 weeks postinjury did not differ between sham and TBI animals regardless of epilepsy outcome (data not shown). A further methodological limitation is that kinetic modeling for PET quantification could not be performed due to the lack of a validated reference region, which is difficult to establish in TBI due to the widespread and heterogeneous nature of TSPO expression. Therefore, %ID was used as a robust measure of tracer uptake. Nevertheless, the potential contribution of differences in tracer kinetics and availability to the observed PET signal remains to be determined.

Together, our findings reveal distinct post‐TBI neuroinflammatory profiles and demonstrate capacity of TSPO PET to predict PTE risk. However, the nonlinear relationship between TSPO expression and epileptogenesis, reflected by lower [^18^F]‐PBR06 binding in PTE^+^ rats, suggests that elevated TSPO signal does not necessarily correspond to greater epilepsy severity or progression. This paradox could be attributed to an insufficient or maladaptive immune response, a shift toward microglial phenotypes not readily detectable by TSPO PET, altered cholesterol metabolism, or contributions from other cellular sources influencing TSPO expression. Therefore, although TSPO PET demonstrated predictive value in the present study, its interpretation as a standalone biomarker remains complex. Future studies integrating multimodal imaging and quantitative lesion analysis, while accounting for systemic and metabolic influences on tracer availability, are warranted to enhance the predictive and translational utility of TSPO PET.

### 4.2 Incidence of PTE was not associated with behavioral impairments

Consistent with previous studies in rodent TBI models, our findings demonstrate persistent cognitive and neurobehavioral impairments following injury.^36^, ^46^, ^47^ Similar deficits have also been reported in both experimental epilepsy models and clinical populations,^4^, ^48^ highlighting shared pathophysiological mechanisms underlying posttraumatic and epileptic comorbidities. However, in our study, behavioral outcomes did not differ between TBI rats that developed PTE and those that remained seizure‐free, which aligns with prior preclinical reports,^49^, ^50^ indicating that the emergence of cognitive and behavioral impairments after TBI does not necessarily associate with epilepsy development.

Although epilepsy has often been considered a major contributor to neurobehavioral comorbidities,^51^ emerging evidence suggests a more complex, bidirectional relationship in which cognitive and affective disturbances may precede and potentially influence epilepsy development and outcome. Clinical studies have shown that mood disorders such as depression can occur prior to epilepsy diagnosis,^52^ and cognitive impairments may also be present before seizure onset, complicating the causal relationship between these conditions.^53^ These findings suggest that behavioral deficits may reflect broader injury‐induced network dysfunction rather than seizure effects. It is also possible that the behavioral measures assessed in this study, although sensitive to TBI‐related alterations, are not sufficiently specific to distinguish animals that later develop epilepsy. Alternatively, epileptogenesis and behavioral dysfunction may arise through overlapping but distinct mechanisms, with different temporal and regional progression. Therefore, future studies should include behavioral assessments at multiple postinjury time points, to determine whether impairments are linked to TBI, epileptogenesis, or both. Clinical evidence indicates that only a subset of TBI patients develop long‐term neurobehavioral deficits, with outcomes influenced by injury characteristics, demographic variables, and neuropsychological factors.^54^ To account for this heterogeneity in our behavioral data, we applied a data‐driven cut‐point approach^50^, ^55^ using sham performance as a reference to define behavioral impairment across tests. Although behavioral impairments were common following TBI, the proportion of rats with impairments did not differ significantly between those that developed epilepsy and those that did not. These observations suggest behavioral impairments after TBI are not reliably associated with PTE. This further supports the idea that behavioral deficits and epilepsy may be driven by partially overlapping yet distinct pathophysiological mechanisms.

### 4.3 TSPO PET as biomarker of neurobehavioral impairments post‐TBI

TBI‐BI^+^ rats exhibiting anxietylike behavior showed increased cortical and thalamic TSPO PET uptake, suggesting that regionally specific neuroinflammation may contribute to the development of certain behavioral impairments following TBI. ROC analysis of cortical [^18^F]‐PBR06 %ID successfully distinguished behaviorally impaired from unimpaired animals, underscoring the potential of TSPO PET as an early biomarker of chronic functional deficits after TBI. These findings align with previous investigations in a preclinical controlled cortical impact model, demonstrating a relationship between TSPO PET, microstructural white matter changes detected via diffusion tensor imaging, and chronic impairments in sensorimotor and cognitive performance.^36^ Similarly, our previous magnetic resonance spectroscopy study revealed distinct metabolic alterations predictive of behavioral outcomes following preclinical TBI,^30^ reinforcing the translational value of multimodal in vivo imaging approaches as noninvasive markers of chronic neurobehavioral sequelae of TBI.

To further explore this relationship, TBI rats were stratified based on the development of impairment across multiple cognitive and affective domains. PCA revealed distinct clustering of the different groups, as rats with multidomain impairments exhibited greater neuroinflammation evidenced by enhanced [^18^F]‐PBR06 %ID as compared to rats with impairments in only one behavioral domain. Our findings are consistent with previous preclinical and clinical TBI studies linking persistent neuroinflammation to long‐term cognitive and neuropsychiatric TBI sequelae.^24^, ^56^ For instance, a study in a rat model of mild fluid percussion injury reported that reactive astrocytosis and neuroinflammation across neocortical regions were associated with impaired spatial learning and altered anxietylike behavior.^57^ Similarly, a recent transdiagnostic meta‐analysis of 156 TSPO PET studies reported modest but significant increases in TSPO binding within corticolimbic circuits across disorders characterized by mood and cognitive dysfunction.^58^

Collectively, these findings support a role for neuroinflammation in post‐TBI behavioral impairments and highlight the utility of TSPO PET as an in vivo imaging tool for predicting chronic functional impairments and the neurobiological burden of TBI.

## 5 CONCLUSIONS

TSPO PET revealed distinct neuroinflammatory profiles following TBI and demonstrated potential as a predictive biomarker for both epileptogenesis and behavioral impairments. Reduced TSPO binding predicted later epilepsy development, whereas elevated thalamic binding was associated with greater behavioral dysfunction. These findings support TSPO PET as a potential tool for identifying individuals at risk for PTE and chronic TBI‐related functional deficits, and further highlight its potential to inform early, targeted therapeutic interventions.

## AUTHOR CONTRIBUTIONS

*Concept and study design*: Idrish Ali as principal investigator and Mohit Javalgekar as project lead, supported by Stefanie Dedeurwaerdere and all authors. *Experimentation*: Mohit Javalgekar, Idrish Ali, Juliana Silva, Peravina Thergarajan, Emma Braine, David K. Wright, Bianca Jupp, and Robert Brkljača. *Data analysis*: Mohit Javalgekar, Idrish Ali, David K. Wright, Bianca Jupp, Lucy Vivash, and Nigel C. Jones. Mohammad Haskali and Usama Kamil supported radiotracer synthesis, and Pablo M. Casillas‐Espinosa provided access to EEG analysis tool. *Data interpretation*: All authors. *Manuscript writing, editing, and reviewing*: Mohit Javalgekar, Idrish Ali, Bianca Jupp, Lucy Vivash, Terence J. O'Brien, Nigel C. Jones and Stefanie D with input from all authors.

## FUNDING INFORMATION

The study was funded by US Department of Defense grant W81XWH‐21‐1‐0927, Alfred Foundation Philanthropic support, and NHMRC Investigator Grants APP1176426 and APP2034258.

## CONFLICT OF INTEREST STATEMENT

The authors declare no competing interests related to the current work. We confirm that we have read the Journal's position on issues involved in ethical publication and affirm that this report is consistent with those guidelines.

## Supporting information


**TABLE S1.** Two‐way repeated‐measures analysis of variance results for [^18^F]‐PBR06 uptake in brain regions.
**TABLE S2.** Occurrence and characteristics of spontaneous recurrent seizures across experimental groups.
**FIGURE S1.** Representative electroencephalographic (EEG) traces in posttraumatic epilepsy rats showing recurrent spontaneous seizures recorded during video‐EEG monitoring at 6 months after traumatic brain injury.
**FIGURE S2.** Epilepsy outcomes. (A) Contingency analysis showing proportion of epileptic animals across genders (male vs. female). (B) Comparison of mean seizure duration between male and female epileptic animals. No significant association was found between gender and posttraumatic epilepsy (PTE) development. (C) Bar plot showing the number of animals exhibiting seizure clusters versus those without seizure clusters in the PTE group.
**FIGURE S3.** Comparison of [^18^F]‐PBR06 percentage injected dose (%ID) in posttraumatic epilepsy (PTE)^+^ rats with and without seizure clusters. Regional translocator protein positron emission tomography %ID values are shown in (A) cortex, (B) hippocampus, (C) thalamus, (D) temporal cortex, and (E) ventral hippocampus at 1 and 4 weeks postinjury in epileptic rats without clusters (PTE^+^ noC; *n* = 8) and with clusters (PTE^+^ C; *n* = 3). No significant differences were observed between groups across regions.
**FIGURE S4.** Predictive value of translocator protein (TSPO) positron emission tomography for identifying behavioral impairment in traumatic brain injury (TBI) rats. (A, B) Results of cut‐point analysis: comparison of platform quadrant duration during Morris water maze test. (C–E) Comparison of [^18^F]‐PBR06 percentage injected dose (%ID) between behavior impaired (TBI‐BI^+^; *n* = 46) and nonimpaired (TBI‐BI^−^; *n* = 15) rats based on cognitive behavior. (F, G) Results of cut‐point analysis: comparison of percentage immobility time during forced swim test. (H–J) Comparison of TSPO %ID TBI‐BI^+^ (*n* = 50) and TBI‐BI^−^ (*n* = 11) rats based on depressionlike behavior. *p < .05.
**FIGURE S5.** Proportion of rats with and without epilepsy across cognitive and affective tasks. Proportion of behavioral impairment in traumatic brain injury animals with and without epilepsy is shown across behavioral tasks: (A) Morris water maze, (B) open field test, and (C) forced swim test. No significant association was found between cognitive or affective impairment and posttraumatic epilepsy development.
**FIGURE S6.** Correlation analyses between early translocator protein positron emission tomography (TSPO PET) signal and seizure severity. Correlation analyses between 1 week TSPO PET percentage injected dose values from (A) cortex, (B) hippocampus, (C) thalamus, (D) temporal cortex, and (E) ventral hippocampus and mean seizure duration are shown (*r* = −.7 to −.9, *p* < .05).
**FIGURE S7.** Postmortem hippocampal translocator protein (TSPO) mRNA expression across experimental groups. (A) Raw TSPO mRNA counts in postmortem hippocampal tissue from posttraumatic epilepsy (PTE)^−^, PTE^+^, and sham animals (*n* = 11/group). (B) Correlation between TSPO mRNA and mean seizure duration in PTE^+^ animals. Each dot represents an individual animal. No significant differences in TSPO expression were observed between experimental groups, and no significant correlation was observed between TSPO counts and mean seizure duration.
**FIGURE S8.** Region of interest (ROI) delineation and representative magnetic resonance imaging (MRI)/positron emission tomography (PET) images following traumatic brain injury (TBI). (A) Representative coronal MRI image showing bilateral volumes of interest delineated using PMOD 4.0. (B) Coregistered [^18^F]‐PBR06 PET/MRI image showing regional tracer uptake within the analyzed ROIs. Representative MRI images from sham, posttraumatic epilepsy (PTE)^−^, and PTE^+^ animals at 7 days (C–E) and 28 days (F–H) post‐TBI are shown. Red arrows indicate the injury site and associated structural alterations.

## Data Availability

The data that support the findings of this study are available from the corresponding author upon reasonable request.
