# Supplementary material for: Translocator protein positron emission tomography as a biomarker for posttraumatic epilepsy and behavioral comorbidities following traumatic brain injury
Source: Epilepsia. 2026 Jul 28;67(11):6047–60. doi: 10.1002/epi.70410 (PMC13768935; doi:10.1002/epi.70410)
Supplement: Supplementary file 1 [file EPI-67-6047-s001.docx]

**Supplementary information**

# **Table 1. Two-way repeated-measures ANOVA results for [18F]-PBR06 uptake in brain regions**

| **Brain region** | **Group effect (Sham vs PTE⁺ vs PTE⁻)** | **Time effect (1 vs 4 weeks)** | **Group × Time interaction** |
| --- | --- | --- | --- |
| **Cortex** | F (2, 69) = 24.90  *P* < 0.001 | F (1, 62) = 74.24  *P* < 0.001 | *P* = 0.253 |
| **Dorsal hippocampus** | F (2, 67) = 17.04  *P* < 0.001 | F (1, 60) = 7.475  *P* = 0.008 | *P* = 0.153 |
| **Thalamus** | F (2, 68) = 22.66  *P* < 0.001 | F (1, 61) = 0.3859  *P* =0.537 | *P* = 0.035 |
| **Ventral hippocampus** | F (2, 69) = 10.53  *P* < 0.001 | F (1, 62) = 3.448  *P* = 0.068 | *P* = 0.560 |
| **Temporal cortex** | F (2, 69) = 6.148  *P* = 0.003 | F (1, 62) = 27.16  *P* < 0.001 | *P* = 0.617 |

**Table 1.** ^a^ Two-way repeated-measures ANOVA of regional [¹⁸F]-PBR06 uptake (%ID) with **group** (Sham, PTE⁺, PTE⁻) and **time** (1 and 4 weeks post-TBI) as factors.

^b^ Main group effects are reported as F values with degrees of freedom and P values are shown;

^c^ P < 0.05 was considered significant.

**Figure 1- Representative EEG traces during a seizure in PTE rats**

**A)**

***Fig 1*** ***Representative EEG traces in PTE rats*** *showing recurrent spontaneous seizures recorded during video-EEG monitoring at 6 months post-TBI.*

**Table I1. Occurrence and characteristics of spontaneous recurrent seizures across experimental groups**

| **Experimental Group** | **Animals with PTE (n)** | **Total Number of Seizures (Mean ± SEM)** | **Average Seizure Duration (s, Mean ± SEM)** |
| --- | --- | --- | --- |
| **Sham Control** | 14 | 0 ± 0 | 0 ± 0 |
| **PTE^-^** | 50 | 0 ± 0 | 0 ± 0 |
| **PTE^+^** | **11** | 2.4 ± 0.54 | 29.19 ± 5.27 |

**Table I1. ^a^** Summary of spontaneous recurrent seizure occurrence and seizure characteristics across experimental groups.

^b^ Data are presented as mean ± SEM.

**Figure 2- Epilepsy outcomes**

**A)	B)**

**C)**

***Fig 2*** *A) Contingency analysis showing* ***proportion of epileptic animals across genders (male vs female) B) comparison of mean seizure duration between male and female epileptic animals.*** *No significant association was found between gender and PTE development.* *C) Bar plot showing the number of animals exhibiting seizure clusters versus those without seizure clusters in the PTE group.*

**Figure 3- Comparison of [18F]-PBR06 %ID in PTE⁺ rats with and without seizure clusters**

***Fig 3 Comparison of [18F]-PBR06 % injected dose (%ID) values between PTE⁺ rats with and without seizure clusters.*** *Regional TSPO-PET %ID values in (A) cortex, (B) hippocampus, (C) thalamus, (D) temporal cortex, and (E) ventral hippocampus at 1 and 4 weeks post-injury in epileptic rats without clusters (PTE⁺ noC; n = 8) and with clusters (PTE⁺ C; n = 3). No significant differences were observed between groups across regions.*

**Figure 4- Predictive value of TSPO PET for identifying behavioural impairment in TBI rats**

***Fig 4*** *A-B) Results of cut point analysis - comparison of platform quadrant duration during Morris water maze test. C-E) Comparison of [****18F]-PBR06*** *% ID between behaviour impaired (TBI-BI^+^, n=46) and non-impaired (TBI-BI^–^, n=15) rats based on cognitive behaviour. F-G) Results of cut point analysis - comparison of % immobility time during forced swim test. H-J) Comparison of TSPO % ID TBI-BI^+^ (n=50) and TBI-BI^-^ (n=11) rats based on depression like behaviour. *p<0.05.*

**Figure 5- Proportion of rats with and without epilepsy across cognitive and affective tasks**

**C)**

**B)**

**A)**

***Fig 5*** ***Proportion of behavioural impairment in TBI animals with and without epilepsy*** *across behavioural tasks: (A) Morris water maze, (B) Open field test, and (c) Forced swim test. No significant association was found between cognitive or affective impairment and PTE development.*

**Figure 6- Correlation analyses between early TSPO PET signal and seizure severity**

***Fig 6-*** *Correlation analyses between 1 week TSPO PET %ID values from (A) cortex, (B) hippocampus, (C) thalamus, (D) temporal cortex, and (E) ventral hippocampus and mean seizure duration (r= -0.7 to -0.9, p<0.05).*

Correlation analyses between early TSPO PET signal and epilepsy outcome measures revealed a significant negative association between 1-week TSPO %ID values and mean seizure duration across relevant brain regions, including cortex (r=-0.91, p=0.001), dorsal hippocampus (r=-0.74, p=0.03), temporal cortex (r=-0.77, p=0.02), and ventral hippocampus (r=-0.79, p=0.03)(Fig 6 A-E). These results further suggest that reduced early TSPO signal was associated with greater epilepsy severity.

**Figure 7- Post mortem hippocampal TSPO mRNA expression across experimental groups**

***Fig 7- A)*** *Raw TSPO mRNA counts in post mortem hippocampal tissue from PTE^-^, PTE^+^ and sham animals (n=11/group). B) Correlation between TSPO mRNA and mean seizure duration in PTE^+^ animals. Each dot represents an individual animal. No significant differences in TSPO expression were observed between experimental groups and no significant correlation was observed between TSPO counts and mean seizure duration.*

**Figure 8- ROI delineation and representative MRI/PET images following TBI**

***Fig 8-*** *A)* *Representative coronal MRI image showing bilateral volumes of interest (VOIs) delineated using PMOD 4.0 B) Co-registered [18F]- PBR06 PET/MRI image showing regional tracer uptake within the analysed ROIs. Representative MRI images from sham, PTE^-^ and PTE^+^ animals at 7 days (C-E) and 28 days (F-H) post TBI are shown. Red arrows indicate the injury site and associated structural alterations.*

**Hippocampal mRNA sequencing**

Following completion of video-EEG monitoring, animals were euthanized and hippocampal tissue was collected for mRNA sequencing. RNA samples were quantified using a Qubit 3.0 fluorometer and libraries were prepared from 300 ng RNA using the NEBNext Ultra II RNA Library Prep Kit for Illumina with NEBNext Poly (A) mRNA Magnetic Isolation Module (E7490) from New England Biolabs. Libraries were quality controlled using an Agilent TapeStation 4200 and sequenced on an Illumina NovaSeq 6000 platform using paired-end 150 bp reads. For the purposes of the present study, TSPO mRNA expression values were extracted from the sequencing dataset and compared between sham, epileptic, and non-epileptic TBI animals, and their relationship with epilepsy outcomes was evaluated.

**Behavioural testing**

**Morris water maze**

The Morris water maze was used to assess cognitive function and spatial learning in rats.^21^ A circular tank (170 cm diameter) contained water at 25 ± 1 °C and platform which is either visible (day 1) or hidden (days 2-6). During training (days 1-6), rats completed four randomized trials daily to locate the platform, guided by fixed visual cues in the tank's quadrants. On the 7th day, a probe trial without the platform evaluated memory retention. Metrics including time, distance, and time spent in the platform quadrant were recorded using TopScan (CleverSys Inc.) software.

**Novel object recognition test**

The novel object recognition test (NORT) was carried out as per the protocol described previously.^22^ Briefly, after a 3-day habituation in arena, rats first explored two identical objects placed within the arena for three minutes. Later, one object was replaced with a novel object, and exploration of both objects was recorded by the overhead camera for another three minutes and analysed using TopScan software. The percentage time spent exploring the novel (TN) and familiar (TF) objects as well as the discrimination index (DI) was calculated as [(TN-TF) / (TN+TF)] × 100 to assess object preference.

**Sucrose preference test**

To investigate depression-like behaviour, the sucrose preference and forced swim tests were conducted as described previously.^17^ The sucrose preference test involved offering rats two pre-weighed drinking bottles, one with 1% sucrose solution and the other with water, for 24 hours. The bottles were re-weighed to assess consumption, and their positions were swapped to eliminate any potential bias. The total amount of fluid consumed from both bottles was measured again 24 hours after switching their positions. Sucrose preference was then calculated as the percentage of sucrose intake relative to total fluid intake. Eight TBI and three sham rats were excluded from the study due to inaccurate initial sucrose concentration in the experiment.

**Forced swim test**

During the forced swim test, rats were allowed to swim for 15 min in a clear glass cylinder (diameter 20 cm) filled with water (25 ± 1.0 °C) to a depth of 30 cm during training period followed by a 5 min trial on the next testing day. The test session was recorded using a side-view video camera, and the total immobility duration during which the rats did not actively attempt to escape the cylindrical water jars was calculated and reported. To minimize bias in behavioural assessments, forced swim test outcomes which rely on subjective interpretation were evaluated by an individual blinded to the experimental conditions.

**Open field & elevated plus maze test**

Anxiety-like behaviour was assessed using open field maze and elevated plus maze as previously described.^17^ The rats were placed in the center of the circular open arena (diameter 100 cm, 40 cm high wall) and allowed to explore the arena for 10 min. The percentage of time spent in the center zone and peripheral zone as well as total distance travelled were calculated after a single ten-minute trial. The elevated plus maze consisted of two open and two closed arms, 110 cm long and 12 cm wide, perpendicular to each other and dividing each in half to form a cross. 50 cm high walls enclosed the closed arms while the open arms contained no walls. During a single ten-minute trial, rats were first placed in the center of the maze and were then free to explore its arms for the duration of the test. The test sessions were recorded by the overhead camera and analysed by the TopScan software for behavioural tracking. Both the total number of entries into the closed and open arms, the percentage of time spent in the open and closed arms and total distance travelled were calculated. The experimental animals underwent behavioural testing in a randomized order.
